# Status of epilepsy care delivery and referral in clinics, hospitals, and epilepsy centers in Japan: A nationwide survey

**DOI:** 10.1002/epi4.12874

**Published:** 2023-12-09

**Authors:** Kentaro Tokumoto, Kiyohito Terada, Norihiko Kawaguchi, Takuji Nishida, Mitsuhiko Yamano, Tomoo Aoyagi, Yuji Tadokoro, Naotaka Usui, Yushi Inoue

**Affiliations:** ^1^ National Epilepsy Center National Hospital Organization Shizuoka Institute of Epilepsy and Neurological Disorders Shizuoka Japan; ^2^ Yokohama Minoru Epilepsy and Developmental Clinic Yokohama Kanagawa Japan; ^3^ Department of General Medicine Tokai University School of Medicine Isehara Kanagawa Japan; ^4^ Department of Neurology Tokai University Hospital Isehara Kanagawa Japan; ^5^ Cocorport Consultation Support Room in Kawasaki Business Division of Comprehensive Support Cocorport Incorporated Kawasaki Kanagawa Japan; ^6^ Japan Epilepsy Association Tokyo Japan

**Keywords:** comprehensive epilepsy care, downward referral, epilepsy surgery, stagnation, upward referral

## Abstract

**Objective:**

Collaboration among medical facilities is crucial to deliver comprehensive epilepsy care to a diverse and large population of people with epilepsy. We conducted a survey among medical facilities of various sizes throughout Japan to investigate the status of epilepsy care delivery, functioning, and referral.

**Methods:**

With the cooperation of the Japan Neurological Society (1428 facilities), Japanese Neurosurgical Society (3489 specialists), and Epilepsy Care Network (948 facilities), a questionnaire was mailed to 5865 locations that provide epilepsy care in Japan. The facilities were classified into clinics (19 beds or less), small hospitals (SH, 20–199 beds), large hospitals (LH, 200 beds or more), and epilepsy centers (EC). The status of epilepsy care delivery, functioning, and referral was compared among the four groups.

**Results:**

Responses were received from 1014 facilities (17.3% response rate). After excluding duplicate responses, 957 facilities were analyzed (394 clinics, 149 SH, 388 LH, 26 EC). EC responded “manageable” in more items of epilepsy care functions in general, especially those related to epilepsy surgery, compared to LH with similar facility size. However, EC responded being less manageable in psychiatric service (61.5%), dietary therapy (46.2%), rehabilitation (53.8%), and patient employment support (61.5%). The percentage of facilities that responded “always able to refer” was highest in clinics (67.6%) and the lowest in EC (40%). Referral difficulties were more commonly encountered in EC, and less common in clinics. In EC, the most common reason for inability to refer was patient or family refusal (64%).

**Significance:**

We have clarified the epilepsy care delivery, functioning, and referral in facilities of various sizes in Japan. This study highlights the issues of downward referral and patient stagnation in EC, which have not received much attention.

**Plain Language Summary:**

A nationwide survey of healthcare facilities ranging in size from small clinics to large hospitals in Japan examined medical care delivery and patient referrals related to epilepsy. Compared to other facilities, epilepsy centers provided a variety of medical services to people with epilepsy but were inadequate in addressing psychiatric symptoms, providing dietary therapy, rehabilitation, and patient employment support. Referrals from epilepsy centers to other medical facilities were often refused by patients and their families. This results in patient crowding at epilepsy centers

**Key points**

- This nationwide survey reveals the current status of epilepsy care delivery, functioning, and referral across a wide range of facility sizes in Japan.
- Epilepsy centers were highly proficient in providing surgery care but not enough in delivering psychiatric service, dietary therapy, rehabilitation, and employment support for patients.
- Epilepsy centers were most likely, while clinics were least likely to have difficulties with referring patients to other facilities.
- The most common reason for epilepsy centers not able to make referral was refusal by the patient or family.
- The issues of downward referral and patient stagnation in epilepsy centers are highlighted.

## 1 INTRODUCTION

Epilepsy is one of the most common chronic neurological disorders, with an estimated prevalence of 0.64% worldwide.^1^ Approximately 60% of people with epilepsy (PWE) may become seizure‐free with appropriate use of antiseizure medication (ASM).^2^, ^3^ In addition, good seizure outcome has been demonstrated in patients with drug‐resistant epilepsy who are surgical candidate.^4^ The quality of life (QOL) of PWE is related to other factors besides seizure control,^5^, ^6^ and comprehensive epilepsy care including psychological treatment, dietary therapy, rehabilitation, support for schooling and employment, disease education, and enlightenment activities is expected to improve QOL, regardless of surgical indication. Even in those whose seizures have been controlled, PWE may have comorbidities, medication‐related adverse effects, concern about marriage or childbirth, social constraints, and stigma, all of which are targets of comprehensive epilepsy care. To deliver comprehensive epilepsy care to a diverse and large population of PWE, network systems among multiple medical facilities and patient referral are necessary.

Previous studies that examined referral between medical facilities focused on referral from primary or secondary care to tertiary care facilities or epilepsy centers for surgical treatment of drug‐resistant epilepsy, and underutilization of surgical treatment has been identified as an issue.^7^, ^8^, ^9^ Several surveys were conducted in physicians, patients, and their families on knowledge of and attitude toward epilepsy surgery.^10^, ^11^, ^12^, ^13^ Barriers to referral to epilepsy surgery included misconceptions about epilepsy surgery by the referring physicians, patients, or families. Some studies also examined the epilepsy care functions in medical institutions at national or regional levels but only focused on tertiary care facilities or epilepsy centers.^14^, ^15^ There is a paucity of nationwide study examining epilepsy care coordination that encompasses medical facilities with diverse sizes ranging from clinics to epilepsy centers.

In order to make future policy decisions, it is necessary to understand the current status and functions of a wide range of medical facilities, but such basic data is lacking. In this study, we surveyed medical facilities of various sizes throughout Japan to examine the state of epilepsy care delivery and functioning, as well as the current status and issues of referral for epilepsy care collaboration.

## 2 MATERIALS AND METHODS

### 2.1 Survey

With the cooperation of the Japanese Society of Neurology (1428 accredited and cooperating facilities), Japan Neurosurgical Society (3489 board‐certified neurosurgeons), and Epilepsy Care Network (948 registered facilities), a questionnaire was sent to 5865 locations providing epilepsy care in Japan during February and March 2018. The survey questionnaire consisted of 21 items in total: (1) profile and epilepsy care capacity of the facility; 6 items, (2) manageability of epilepsy care functions; 1 item, (3) current status of referral and epilepsy care collaboration; 2 items, and (4) opinion about the development of epilepsy practice coordination system; 12 items. In the present report focusing on the status and issues of epilepsy care and referral, only items from (1) to (3) were analyzed, while (4) “opinions about the development of the epilepsy care coordination system” was not included in analysis because it is outside of the scope of this report. Details of the questionnaire are presented in Data S1. The questions on the manageability of epilepsy care functions were responded by choosing one out for four options: “manageable”, “partially manageable”, “partially unmanageable”, and “unmanageable”.

In this study, an epilepsy center that was assumed to provide tertiary or quaternary epilepsy care was defined as a member institution of the Japan Epilepsy Center Association (JEPICA). The medical facilities were divided into four groups: clinics were defined as 19 beds or fewer, small hospitals (SH) as 20 to 199 beds, large hospitals (LH) as 200 or more beds (not JEPICA member institutions), and epilepsy centers (EC). All the EC were facilities with 200 beds or more and were not included in LH, and EC were analyzed independently.

JEPICA consists of 36 epilepsy centers. Three of these facilities are associate facilities (facilities that intend to join but have not yet met the criteria of an epilepsy center). The remaining 33 facilities meet the criteria of epilepsy center. The criteria include the deployment of epileptologists and epilepsy‐specific social worker personnel, a system for providing acute care for conditions such as epileptic seizures and status epilepticus, drug therapy and therapeutic drug monitoring, epilepsy monitoring unit, presurgical evaluation, epilepsy surgery, and community collaboration capabilities. One epilepsy center of JEPICA which is composed of multiple facilities (two university hospitals and one clinic) was not included in the EC group in this study, but the three facilities was classified individually and analyzed.

### 2.2 Statistical analysis

Comparisons among four groups (clinics, SH, LH, and EC) were conducted using the Kruskal–Wallis test for discrete variables (number of patients or surgeries) and the chi‐square test for categorical variables (status of epilepsy care function and referral). The Bonferroni method was used to correct for multiple comparisons. For epilepsy care functions, the numbers of facilities that responded “manageable” were compared. Facilities with missing data were excluded from each analysis. Results were considered statistically significant for two‐sided *P* < 0.05. All statistical computations were performed using SPSS 25.0 for Windows (SPSS Inc.).

### 2.3 Institutional approval

The study protocol was approved by ethics committees of NHO Shizuoka. Institute of Epilepsy and Neurological disorders (no. 2018‐10).

## 3 RESULTS

Among 5865 facilities to which the questionnaire was sent, 1014 facilities responded (17.3% response rate). After excluding duplicate responses, we finally analyzed the responses from 957 facilities. The total number of facilities analyzed were 394 clinics, 149 SH, 388 LH, and 26 EC.

### 3.1 Capacity and medical service provision in participating facilities

Table 1 shows the deployment of specialists for epilepsy care and medical equipment at the participating facilities. Neurosurgeons were the most common specialist engaged in epilepsy care, regardless of facility size, and psychiatrists were the least common. Magnetic resonance imaging (MRI) was highly available, with rates of 35.6% in clinics, 69.4% in SH, 89.9% in LH, and 100% in EC. Three tesla‐MRI was installed in 45.5% of LH and 88.5% of EC. The availability rate of digital electroencephalography (EEG) was 25.4% in clinics, 50.3% in SH, 69.7% in LH, and 96.2% in EC. Irrespective of facility size, the availability rate of digital EEG was more than two‐fold that of analog EEG. Clinics showed particularly low access to EEG (10.3% analog, 25.4% digital) and relatively better access to diagnostic imaging such as computed tomography (45.9%) and MRI (35.6%). Long‐term EEG was not available in the majority of facilities, except for EC (92.3%). Magnetoencephalography (MEG) and positron emission tomography (PET) were installed in 38.5% and 61.5% of EC, respectively, but available in less than 20% in other facilities.

**TABLE 1:** Deployment of specialists responsible for epilepsy care and medical equipment in the participating facilities.

	Clinics (*n* = 394)	Small hospitals (*n* = 149)	Large hospitals (*n* = 388)	Epilepsy centers (*n* = 26)
Specialists				
Pediatricians	40 (10.2%)	35 (23.5%)	261 (62.3%)	24 (92.3%)
Neurologists	132 (33.5%)	74 (50.0%)	286 (73.7%)	23 (88.5%)
Neurosurgeons	207 (52.5%)	87 (58.4%)	289 (74.5%)	25 (96.1%)
Psychiatrists	40 (10.2%)	19 (12.8%)	144 (37.1%)	18 (69.2%)
Others	14 (3.6%)	14 (9.4%)	18 (4.6%)	4 (15.4%)
Equipment				
CT	179 (45.9%)	137 (93.2%)	383 (99.0%)	26 (100%)
MRI	139 (35.6%)	102 (69.4%)	348 (89.9%)	26 (100%)
3 Tesla‐MRI	9 (2.3%)	25 (17.0%)	176 (45.5%)	23 (88.5%)
Functional MRI	3 (0.77%)	13 (8.8%)	71 (18.3%)	20 (76.9%)
SPECT	4 (1.0%)	20 (13.6%)	271 (70.0%)	25 (96.1)
Analogue EEG	40 (10.3%)	39 (26.5%)	122 (31.5%)	9 (34.6)
Digital EEG	99 (25.4%)	74 (50.3%)	270 (69.8%)	25 (96.2%)
Long‐term EEG	9 (2.3%)	14 (9.5%)	78 (20.1%)	24 (92.3%)
MEG	0 (0%)	0 (0%)	13 (3.4%)	10 (38.5%)
PET	4 (1.0%)	6 (4.1%)	77 (19.9%)	16 (61.5%)

Abbreviations: CT, computed tomography; EEG, electroencephalography; MEG, magnetoencephalography; MRI, magnetic resonance imaging; PET, positron emission tomography; SPECT, single photon emission computed tomography.

Table 2 shows the numbers of patients and surgeries during the 1‐year period from April 2016 to March 2017. The median numbers of new patients, inpatients, and emergency patients related to epilepsy tended to increase with increase in facility size. However, the largest gap existed between LH and EC: median number of new epilepsy patients were 6.8 times, inpatients were 8.8 times, and emergency patients were four times higher in EC compared to LH. When analyzed statistically, there were significant differences in the numbers of new patients, inpatients, and emergency patients in all two‐group comparisons among clinics, SH, LH, and EC. There were significant differences in resective and disconnection surgery, palliative surgery, intracranial EEG, and other surgeries between EC and clinics, between EC and SH, and between EC and LH, but no significant differences in other comparisons.

**TABLE 2:** Status of epilepsy medical services provided in the participating facilities.

	Clinics	Small hospitals	Large hospitals	Epilepsy centers
New patients/year				
Facilities	340	132	372	26
Median (IQR)	5 (2–10)	10 (3–20)	28 (10–61)	204.5 (121.8–491.8)
Total	4599	3083	23 854	8567
Hospitalized patients/year				
No. of responding facilities	79	113	371	26
Median (IQR)	0 (0)	8 (3–20)	20 (10–50)	175.5 (100.5–309.8)
Total	72	2406	14 287	8690
Emergency patients/year				
No. of responding facilities	72	101	349	26
Median (IQR)	2 (1–4.3)	8 (4–20)	20 (7–60)	80 (30.5–163.5)
Total	282	1917	16 368	3104
Resective or disconnection surgery/year				
No. of responding facilities	186	74	171	24
Median (IQR)	0 (0–0)	0 (0–0)	0 (0–0)	10 (5.5–18.25)
Total	0	2	84	365
Palliative surgery/year				
No. of responding facilities	188	74	167	23
Median (IQR)	0 (0–0)	0 (0–0)	0 (0–0)	5 (1.5–15)
Total	0	0	38	192
Intracranial electrode implantation / year				
No. of responding facilities	188	74	166	23
Median (IQR)	0 (0–0)	0 (0–0)	0 (0–0)	4 (1–8)
Total	0	0	39	148
Other surgeries/year				
No. of responding facilities	394	149	389	26
Median (IQR)	0 (0–0)	0 (0–0)	0 (0–0)	0 (0–2)
Total	0	0	36	50

Abbreviation: IQR, interquartile range.

### 3.2 Manageability of epilepsy care functions

**FIGURE 1:**
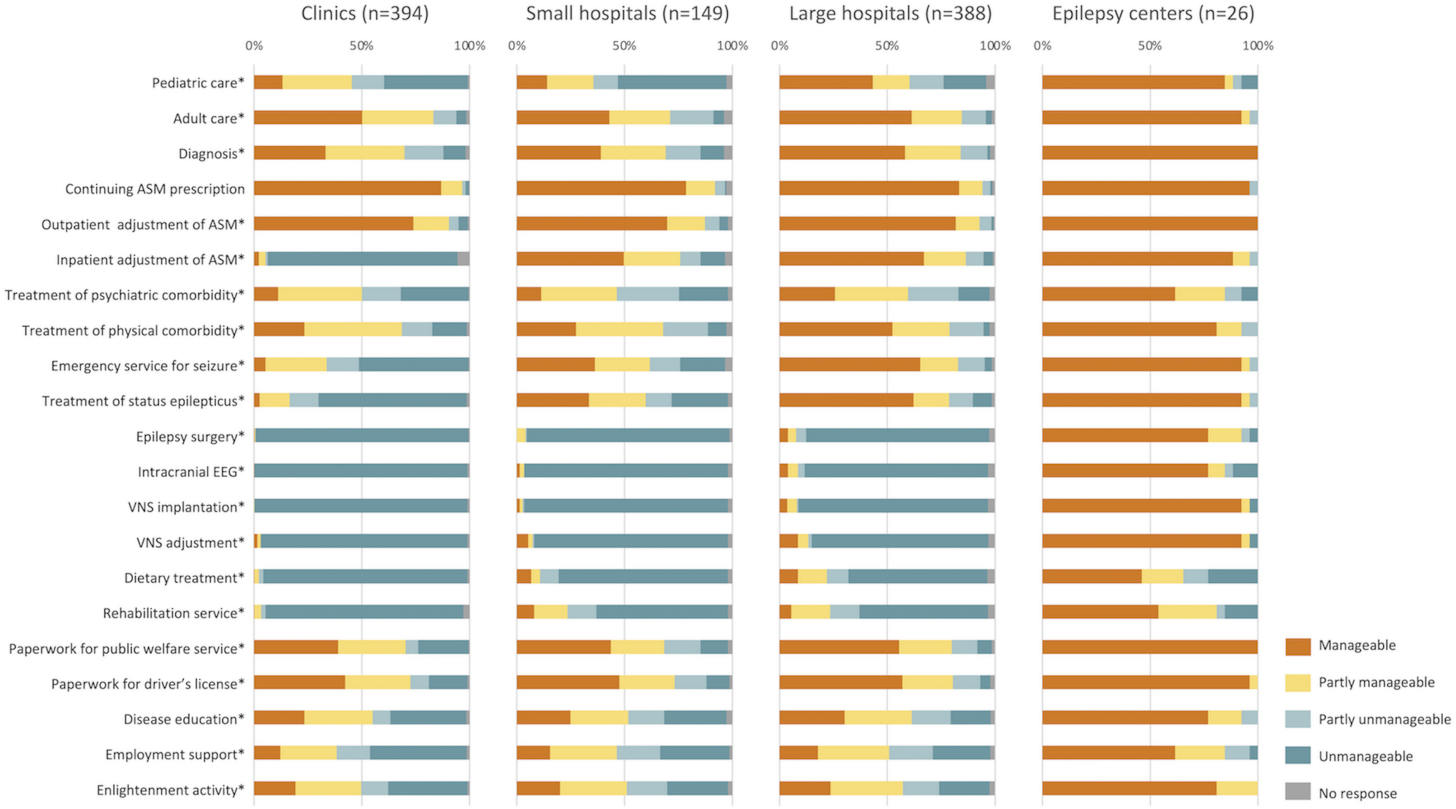
Status of manageability of epilepsy care functions. *Statistically significant different (*P* < 0.05) among four groups when facilities that responded “manageable” were compared. The results of post‐hoc analysis of multiple comparison between two groups are shown in Data S2. ASM, antiseizure medications; EEG, electroencephalography; VNS, vagus nerve stimulation.

Figure 1 shows the status of manageability of epilepsy care functions in the four groups. EC generally had more “manageable” items than other facilities; in particular, EC had markedly more “manageable” items related to epilepsy surgery than LH. This result indicated that epilepsy surgery was rarely performed even in LH. However, although EC were highly proficient in managing epilepsy surgery‐related care, smaller proportions of EC reported “manageable” in the treatment of psychiatric comorbidity (16/26, 61.5%), dietary therapy (12/26, 46.2%), rehabilitation (14/26, 53.8%), and patient employment support (16/26, 61.5%).

Statistical analysis of the numbers of facilities that responded “manageable” in each item showed significant differences among four groups in all the functions except “continuing ASM prescription”. The results of detailed statistical analyses are shown in Data S2.

### 3.3 Current status of referral and epilepsy care collaboration

**FIGURE 2:**
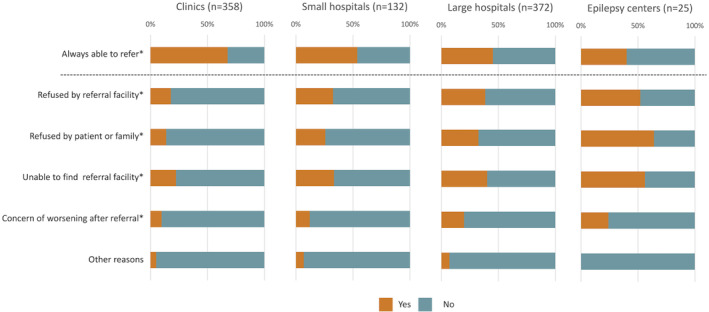
Status of referral to other facilities. The facilities that did not respond to this question (36 clinics, 17 small hospitals, 16 large hospitals, and 1 epilepsy center) were not included in the bar charts. The item above the dashed line is “referral can be made”, and a higher percentage of “yes” response suggests smoother referral. The items below the dashed line are “referral cannot be made for some reasons” and a higher percentage of “yes” response suggests greater difficulty in making referral. Referral tended to be smoother in lower‐level facilities and more difficult in higher‐level facilities. *Statistically significant difference (*P* < 0.05) among four groups. The results of post‐hoc analysis of multiple comparison between two groups are shown in the text.

Figure 2 shows the status of referral to other facilities in the four groups. Responses to this question were received from 358 clinics, 132 SH, 372 LH, and 25 EC. In terms of the status of referral, smaller facilities were more likely to response that they were always able to refer, while larger facilities were more likely to report unable to refer for various reasons (“refused by referral facility”, “refused by patient or family”, or “unable to find referral facility”). In EC, refusal by patient or family was the most common reason that prevented referral to other facilities, and this pattern was observed only in EC. Statistical comparisons among four groups for each item showed the following results. “Always able to refer” and “refused by referral facility” were significantly different between clinics and SH, between clinics and LH, and between clinics and EC, but not significantly different in other comparisons. “Unable to find referral facility” was significant different between clinics and LH and between clinics and EC, but not significantly different in other comparisons. “Refused by patient or family” was not significantly different between SH and LH, but was significantly different otherwise. “Concern of worsening after referral” was significantly different between clinics and LH, but not significantly different in other comparisons. “Other reasons” were not significantly different in all comparisons.

**FIGURE 3:**
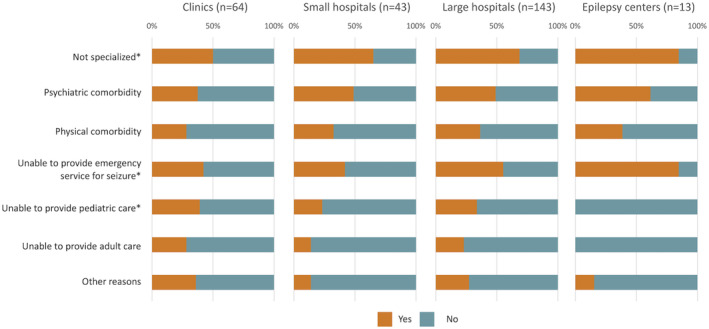
Reasons for refusal by referral facilities. Facilities that did not respond to this question (330 clinics, 106 small hospitals, 245 large hospitals, and 13 epilepsy centers) were not included in the bar charts. *Statistically significant difference (*P* < 0.05) among four groups. The results of post‐hoc analysis of multiple comparison between two groups are shown in the text.

When examining the reasons for refusal by referral facilities (Figure 3), “not specialized in epilepsy” was the most common reason when referral was made in clinics, SH, and LH. When referral was made in EC, the most common reason was “unable to provide emergency care”, followed by “not specialized in epilepsy” and “psychiatric comorbidity”.

The larger the facilities, the higher was the percentage of “yes” response to all the reasons. Notably, there was no refusal due to inability to provide pediatric or adult care when referral was made in EC. Regardless of facility size, refusal due to psychiatric comorbidity was more frequent than refusal due to physical comorbidity. In general, there was little difference in response pattern between SH and LH. Statistical comparisons between groups showed the following results. “Not specialized in epilepsy” was significantly different among four groups overall, but not between two groups. “Psychiatric comorbidity”, physical comorbidity”, “unable to provide adult service”, and “other reasons” were not significantly different among four groups overall. “Unable to provide emergency service” was significantly different between clinics and EC and between SH and EC. “Unable to provide pediatric service” was significantly different only between clinics and EC.

## 4 DISCUSSION

This nationwide cross‐sectional study in Japan provides new data on the epilepsy medical services, status of manageability of epilepsy care functions, and status of referral and care collaboration across a wide range of facility sizes from clinics to LH and EC. Even in EC, provision of care was not enough in the treatment of psychiatric comorbidity, dietary therapy, rehabilitation, and patient employment support. Furthermore, EC have difficulties in referring patients to other facilities.

### 4.1 Status of medical services and epilepsy care functions in responding facilities

EC had 2.2 times more inpatients than emergency patients, while SH and LH had almost the same number of inpatients and emergency patients (Table 2). Although emergency patients are not always admitted, these results suggest a close association between emergency visits and hospitalization (for status epilepticus, trauma related to seizures, and admission for close observation) in SH and LH, and inpatient care as an extension of emergency service. On the other hand, EC had far more inpatients than emergency cases, suggesting that the purposes of hospitalization were more specialized and diverse, such as diagnosis of epilepsy by long‐term EEG, differentiation from related diseases, presurgical evaluation, epilepsy surgery, drug reconciliation, initiation of dietary therapy, and rehabilitation. This finding indicates that advanced epilepsy‐specific care is not widely available outside EC.

EC managed epilepsy surgery‐related care proficiently, but the proportions of facilities reporting “manageable” in treatment of psychiatric comorbidity (61.5%), dietary treatment (46.2%), rehabilitation (53.8%), and employment support (61.5%) were low, and these are the problems facing EC in Japan. Epilepsy is a disorder of the brain characterized not only by seizures, but also by their neurobiological, cognitive, psychological, and social consequences.^16^ Thus, EC need also to perform nonsurgical functions proficiently to provide comprehensive epilepsy care. This survey showed many neurosurgeons engaged in epilepsy care in EC, but few psychiatrists, which may partially explain the pattern of epilepsy care delivery in EC. Furthermore, in Japan, epilepsy surgery and long‐term video‐EEG monitoring are highly reimbursed by health insurance, while other care items such as rehabilitation for PWE are less reimbursed. Some PWE have missed opportunities to receive education and vocational training because of epilepsy. Rehabilitating such individuals and reintegrating them into the society and eventually into employment is expected to improve their QOL as well as benefit the society.^17^, ^18^ To advocate the importance of rehabilitation in planning healthcare policy, data to support the rationale is needed.

Epilepsy surgery, regardless of the type, was performed exclusively in EC and was not performed even in LH. However, in EC, a median of 10 resective or disconnection surgeries were performed in 1 year. In other words, there were many centers that performed less than 10 epilepsy surgeries per year. A volume‐outcome relationship in epilepsy surgery has been reported, suggesting that epilepsy surgery is less likely to be performed in low‐volume hospitals^8^ and that surgery in low‐volume centers increases complications.^19^ These concerns may also apply to some EC in Japan, where the number of surgeries is small. In addition, the small number of surgeries may be associated with small numbers of various health professionals providing epilepsy care, including rehabilitation staff, eventually leading to a low quality of comprehensive epilepsy care delivery.

### 4.2 Current status of referral and epilepsy care collaboration

**FIGURE 4:**
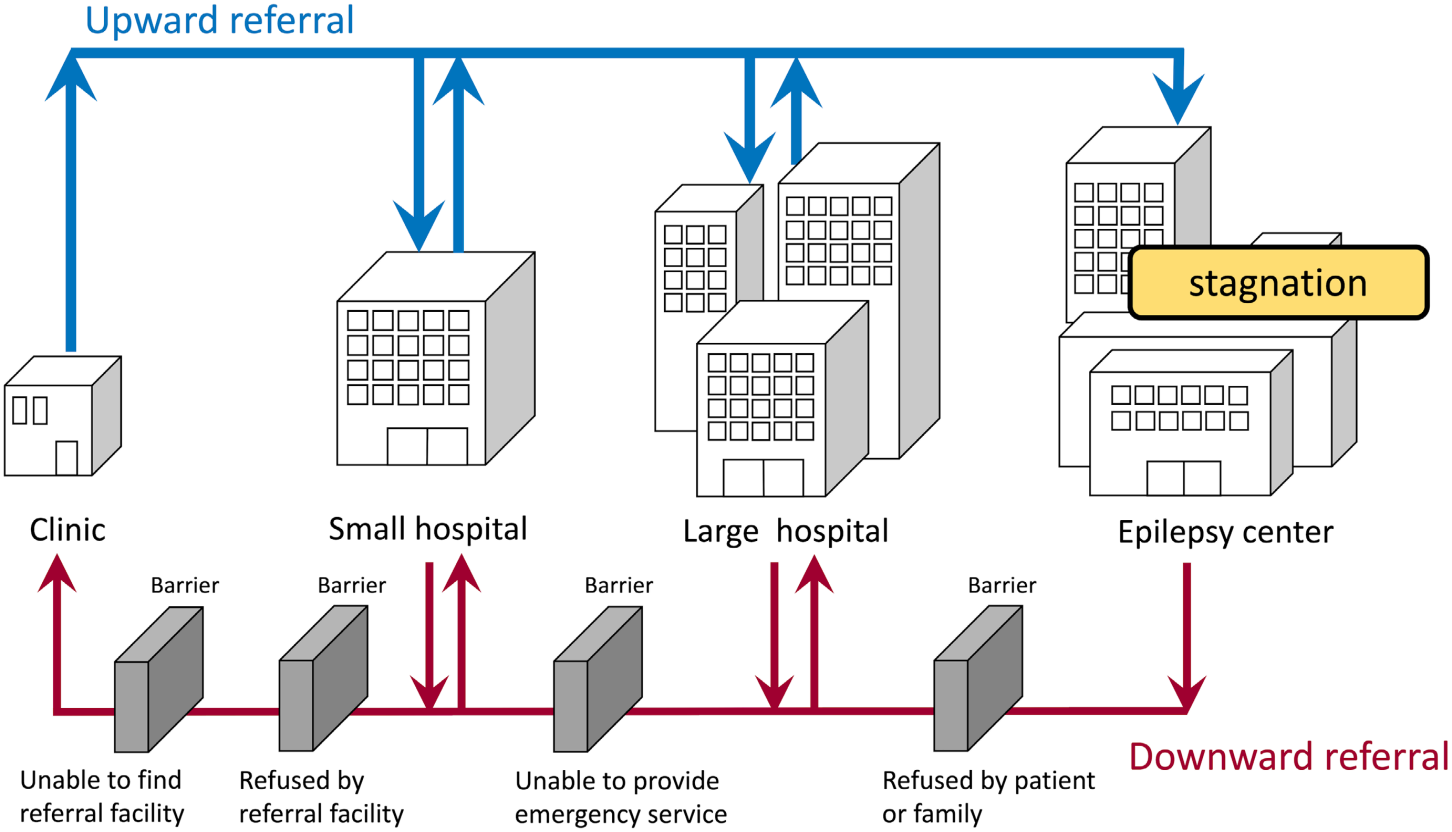
Schematic representation of referral status and barriers to referral made in epilepsy centers. Blue arrow indicates upward referral and red arrow indicates downward referral. “Barrier” denotes a barrier to referral significantly more commonly encountered by epilepsy centers than other facility groups. Barriers to referral with response rates not significantly different between facility groups are not shown. For example, “not specialized in epilepsy” was the second most common reason and “psychiatric comorbidity” the third most common reason for refusal by referral facility, but they are not shown because the response rates were not significantly higher for EC than other facility groups. Refusal because of inability to provide pediatric or adult care was not encountered when referring from epilepsy centers to other facilities.

The proportions of facilities responding “always able to refer” was the largest in clinics (67.6%) and the smallest in EC (40%). The most common reason for unable to refer in EC was “refused by patient or family” (64%). The other reasons cited were “refused by referral facility”, “unable to find referral facility” and “concern of worsening after referral”, all of which were more common among LH and EC and less common among clinics and SH. While previous studies have reported delay in referral to tertiary or quaternary care facilities and underutilization of surgical treatment, this study highlights the difficulties of referral encountered in tertiary care facilities, especially EC (Figure 4). A bidirectional (two‐way) referral system is essential for delivery of comprehensive epilepsy care within the existing healthcare system. Referral from a primary care clinic to a specialty care facility is called upward referral (UR), and vice versa is called downward referral (DR). Both for epilepsy and other diseases, many studies have investigated upward referral, while reports on DR are scarce. Several reports of DR in general medical care in China indicate that DR is a more serious and difficult problem than UR.^20^, ^21^ Patient refusal is a well‐known cause that prevents DR, which is consistent with the results of this study. Patients generally believe that they will receive better medical care in hospitals with better medical equipment. In Japan, all citizens are enrolled in the national health insurance system; medical fees are fundamentally the same in all medical institutions and patients are basically free to visit any facility of their choice. As a result, patients tend to prefer LHs, and crowding in outpatient clinics of higher‐level medical facilities is a frequent and serious problem.^22^ This study shows that epilepsy care is no exception. In Japan, to ease congestion in LHs caused by patients presenting with minor illnesses, a patient who first presents to a LH without referral now has to pay an additional fee, but this does not apply to subsequent visits, and the effectiveness of this measure is limited.

Yu et al.^21^ reported doctor and patient factors that hinder DR in China. Doctors' willingness to accept DR was influenced by attitude toward workload caused by DR, perception of the limitations of health resources in hospitals, and level of education. Thus, well‐educated doctors did not perceive that DR would increase workload, and doctors with a more comprehensive understanding of hospitals and the DR process were more likely to make a DR decision. Patients' willingness increased if DR would save cost. In another study of willingness for DR among hospitalized older patients, Jing et al.^20^ found that 33.4% of patients were willing to accept DR if advised by a physician. Patients' self‐rated health and their experience of treatment in primary care setting also influenced patients' willingness for DR. Doctor's advice may greatly influence a patient's decision regarding DR, and improving the level of care in primary care providers may in turn smooth the process of DR. In a study of factors that hinder referral in epilepsy care in Australia, epilepsy specialists expressed the view that even stable patients prefer to continue going to tertiary epilepsy centers, resulting in overcrowding and delays in accepting new referrals.^23^ Our study supports their view with large‐scale data.

Medical resources are limited, and stagnation of patients at higher‐level epilepsy care facilities deprives the opportunities of current and future patients to receive necessary and timely care. This potential hindrance is “invisible” at the site where stagnation occurs, but if there is a long waiting list for appointment, it may already be a red flag. In order to promote DR, tertiary and quaternary facilities and academic societies need to engage in communication, education, and enlightenment activities to gain the understanding of patients and primary or secondary care facilities. In terms of healthcare policy, measures such as providing additional incentives in insurance reimbursement to further promote DR should be considered. Adoption of new technologies such as decision support systems is also expected to contribute to the promotion of interactive referral.^24^

### 4.3 International comparison of healthcare access and quality

The external validity of the results of this study is ideally examined by conducting an international comparison of the results obtained in Japan with those of other countries. However, such comparison will only be meaningful if the methods of sample collection and analysis are comparable between the countries studied. An international comparison of healthcare access and quality (HAQ) of 32 causes (diseases) for which mortality is amenable to healthcare (death from causes that should not occur in the presence of effective care), including epilepsy, was conducted among 195 countries and territories using the HAQ index.^25^ Japan ranked the 12th highest in HAQ index, indicating that Japan is one of the countries with the highest access to and quality of healthcare in the world. In the comparison of age‐standardized risk‐standardized death rates for individual causes, Japan shared with five other countries in scoring 100 out of 100 for epilepsy, confirming the high‐level performance in epilepsy as in overall healthcare. Notably, Japan also had the smallest disparity in HAQ index among the 47 prefectures. The subnational disparity of this index was more than twice as high in the United States and more than three times as high in England as in Japan, while China and India had even bigger gaps between locations. From these results, it may be speculated that the high accessibility to medical care in Japan may be associated with the difficulties in DR and stagnation of patients in higher tertiary care facilities. These challenges identified in our study may apply equally to countries with good access to healthcare (Europe and its vicinity, Canada, Australia, and New Zealand) and to regions within countries with good access to healthcare; conversely, they may be less applicable to countries with insufficient access to healthcare (Southeast Asia and Africa). Even in countries and national regions where URs are currently low and DRs are not a problem, the same issues may arise in the future as access to healthcare progresses.

### 4.4 Limitations

The survey response rate (17.3%) was not high. Based on the annual reports of the Japan Neurosurgical Society, the total number of epilepsy surgery conducted in Japan between 2015 and 2018 was approximately 1200 per year.^26^ In this study, a total of 954 epilepsy surgeries in 1 year were reported (Table 2), which corresponds to 80% of the 1200 surgeries nationwide. Besides, since the population of Japan in 2016 was 127 million^27^ and the incidence of epilepsy in high‐income countries is 48.86/100 000 person‐year,^1^ the estimated number of new cases of epilepsy in Japan is 62 000 per year. In this study, the total number of new patients in all the responding facilities was 40 103 (Table 2). Although the possibility of duplicate patient counting cannot be ruled out, this number corresponds to 64.6% of the estimated annual number of new cases in Japan. These figures infer that the data of this study were obtained from medical institutions caring for approximately two‐thirds of the total number of epilepsy patients in Japan, which may mitigate the limitation of the low response rate of 17.3%.

It is also possible that the responding facilities represent those with a high interest in epilepsy care. Actually, EC had a high response rate of 74% (26/35). The coverage of pediatric and psychiatric facilities may be relatively low because the questionnaire was sent through the member lists of the Japanese Society of Neurology, Japan Neurosurgical Society, and Epilepsy Care Network. However, LHs and general hospitals with multiple departments were included. Clinics and smaller facilities with pediatric or psychiatric service, which are not registered in Epilepsy Care Network are not covered. Nevertheless, a national survey of this size has not been reported and would yield valuable data. The questionnaire in this study asked whether referring doctors were able to refer when they felt they should. Therefore, this study only partially reflects the commonly cited issue of UR: “referral to EC is not made at the appropriate timing”. However, the results of this study do not negate the problem of UR.

Another limitation is that the numbers of new patients, emergency patients, and inpatients may include patients with acute symptomatic seizures, since most LH in Japan are acute care hospitals with emergency service.

## 5 CONCLUSION

This study clarifies the status of epilepsy care delivery and the challenges encountered across various facility sizes in Japan. The results shed light on the issue of DR that has not received much attention until now, and the need for countermeasures. DR is inextricably linked to UR, and the entire healthcare system will become dysfunctional unless bidirectional patient referral flows smoothly. It is necessary not only to increase the number of epilepsy centers and improve their functions but also to raise the level of epilepsy care and education, including primary care. Communication between patients and doctors as well as between medical facilities in the local community should be enhanced to promote smooth two‐way referral.

## AUTHOR CONTRIBUTIONS

Tokumoto and Terada had full access to all of the data in the study and took responsibility for the integrity of the data and the accuracy of the data analysis. All authors provided the study concept and design, acquisition, analysis, or interpretation of data and critical revision of the manuscript for important intellectual content. Tokumoto, Terada, and Kawaguchi drafted the manuscript. Tokumoto provided the statistical analysis. Terada provided the administrative, technical, or material support. Tokumoto and Terada supervised the study.

## CONFLICT OF INTEREST STATEMENT

None of the authors has any conflict of interest to disclose. We confirm that we have read the Journal's position on issues involved in ethical publication and affirm that this report is consistent with those guidelines.

## ETHICS STATEMENT

The study protocol was approved by ethics committees of NHO Shizuoka Institute of Epilepsy and Neurological disorders (no. 2018‐10).

## Supporting information


Data S1.

## Data Availability

The datasets used and/or analyzed during the current study are available from the corresponding author on reasonable request.
