# Supplementary material for: Status of epilepsy care delivery and referral in clinics, hospitals, and epilepsy centers in Japan: A nationwide survey
Source: Epilepsia Open. 2023 Dec 9;9(1):314–24. doi: 10.1002/epi4.12874 (PMC10839338; doi:10.1002/epi4.12874)
Supplement: Supplementary file 1 — Data S1. [file EPI4-9-314-s001.docx]

**Supplementary Data 1. Survey Questionnaire**

We would like to ask the person responsible for the department in charge of epilepsy care at your institution to answer the following questions.

Q1. Please provide the name, location, and number of beds of your facility.

Q2. Please indicate the number of doctors engaged in epilepsy care at your facility.

| Pediatrician |  |
| --- | --- |
| Neurosurgeon |  |
| Psychiatrist |  |
| Neurologist |  |
| Other |  |

Q3. Please tell us about the examination equipment available at your facility. You may choose any number of them.

| 1. CT 2. MRI 3. 3T- MRI 4. f-MRI 5. SPECT 6. Analogue EEG 7. Digital EEG 8. Long term video-EEG monitoring 9. MEG 10. PET |
| --- |

Q4. Please indicate the number of new patients and hospitalized patients with epilepsy as the chief complaint for the one-year period between April 2016 and March 2017.

| New patients | persons |
| --- | --- |
| Hospitalized patients | persons |

Q5. Please indicate the number of emergency admissions with epileptic seizures as the chief complaint for the one-year period between April 2016 and March 2017.

| Emergency patients | persons |
| --- | --- |

Q6. If your institution performs epilepsy surgery, please indicate the number of epilepsy surgeries for the one-year period between April 2016 and March 2017.

| Resective or disconnection surgery | cases |
| --- | --- |
| Palliative surgery | cases |
| Intracranial electrode implantation | cases |
| Other surgery | cases |

Q7. Is there problem in epilepsy care in your facility? Choose one out of the four options for each item.

|  | Manageable | Partly manageable | Partly unmanageable | Unmanageable |
| --- | --- | --- | --- | --- |
| Pediatric care | 1 | 2 | 3 | 4 |
| Adult care | 1 | 2 | 3 | 4 |
| Diagnosis | 1 | 2 | 3 | 4 |
| Continuing ASM prescription | 1 | 2 | 3 | 4 |
| Outpatient ASM adjustment | 1 | 2 | 3 | 4 |
| Inpatient ASM adjustment | 1 | 2 | 3 | 4 |
| Treatment of psychiatric comorbidity | 1 | 2 | 3 | 4 |
| Treatment of physical comorbidity | 1 | 2 | 3 | 4 |
| Emergency service for seizures | 1 | 2 | 3 | 4 |
| Treatment of status epilepticus | 1 | 2 | 3 | 4 |
| Epilepsy surgery | 1 | 2 | 3 | 4 |
| Intracranial EEG | 1 | 2 | 3 | 4 |
| VNS implantation | 1 | 2 | 3 | 4 |
| VNS adjustment | 1 | 2 | 3 | 4 |
| Dietary treatment | 1 | 2 | 3 | 4 |
| Rehabilitation service | 1 | 2 | 3 | 4 |
| Paperwork for public welfare service | 1 | 2 | 3 | 4 |
| Paperwork for driver’s license | 1 | 2 | 3 | 4 |
| Disease education | 1 | 2 | 3 | 4 |
| Employment support | 1 | 2 | 3 | 4 |
| Enlightenment activity | 1 | 2 | 3 | 4 |

Q8. Please indicate the status of referral from your facility to other hospitals and the reasons for any refusal from referral facility.

|  | Yes | No |
| --- | --- | --- |
| Always able to refer | 1 | 2 |
| Refused by referral facility (Choose the reason in next question) | 1 | 2 |
| Refused by patient or family | 1 | 2 |
| Unable to find referral facilities | 1 | 2 |
| Doctors’ concern of worsening after referral | 1 | 2 |
| Other reasons | 1 | 2 |

Q9. Reasons for refusal by referral facilities.

|  | Yes | No |
| --- | --- | --- |
| Not specialized in epilepsy | 1 | 2 |
| Psychiatric comorbidity | 1 | 2 |
| Physical comorbidity | 1 | 2 |
| Unable to provide emergency  service for seizure | 1 | 2 |
| Unable to provide pediatric care | 1 | 2 |
| Unable to provide adult care | 1 | 2 |
| Other reasons | 1 | 2 |

**Supplementary Data 2. Statistical Analysis of Manageability of Epilepsy Care Functions.**

| **Epilepsy care function** | **Statistical analysis results** |
| --- | --- |
| Pediatric service | Patten A |
| Adult service | Patten A |
| Diagnosis | Patten A |
| Continuing ASM prescription | Not significant |
| Outpatient ASM adjustment | Pattern B |
| Inpatient ASM adjustment | Pattern C |
| Treatment of psychiatric comorbidity | Patten A |
| Treatment of physical comorbidity | Patten A |
| Emergency service for seizures | Pattern D |
| Treatment of status epilepticus | Pattern D |
| Epilepsy surgery | Pattern E |
| Intracranial EEG | Pattern E |
| VNS implantation | Pattern E |
| VNS adjustment | Pattern E |
| Dietary treatment | Pattern F |
| Rehabilitation service | Pattern F |
| Paperwork for public welfare service | Pattern E |
| Paperwork for driver’s license | Pattern E |
| Disease education | Pattern G |
| Employment support | Pattern G |
| Enlightenment activity | Pattern G |

Statistical analysis of the numbers of facilities that responded “Manageable” showed significant differences among four groups in all the functions except “continuing ASM prescription”. Other statistical analysis results are as follows. Pattern A: No significant difference between clinics and SH, but significant differences in other comparisons. Pattern B: No significant difference between clinics and SH and between LH and EC, but significant differences otherwise. Pattern C; No significant difference between LH and EC, but significant differences otherwise. Pattern D; Significant differences in all two-group comparisons. Pattern E; No significant difference between clinics and SH and between SH and LH, but significant differences otherwise. Pattern F; No significant difference between SH and LH, but significant differences otherwise. Pattern G; Significant difference between EC and other groups, but no significant differences otherwise.
